# Surface Plasmon Resonance Sensing Detection of Mercury and Lead Ions Based on Conducting Polymer Composite

**DOI:** 10.1371/journal.pone.0024578

**Published:** 2011-09-09

**Authors:** Mahnaz M. Abdi, Luqman Chuah Abdullah, Amir R. Sadrolhosseini, Wan Mahmood Mat Yunus, Mohd Maarof Moksin, Paridah Md. Tahir

**Affiliations:** 1 Laboratory of Biopolymer and Derivatives, Institute of Tropical Forestry and Forest Products (INTROP), Universiti Putra Malaysia, Serdang, Selangor, Malaysia; 2 Department of Physics, Faculty of Science, Universiti Putra Malaysia, Serdang, Selangor, Malaysia; Joint Research Centre - European Commission, Germany

## Abstract

A new sensing area for a sensor based on surface plasmon resonance (SPR) was fabricated to detect trace amounts of mercury and lead ions. The gold surface used for SPR measurements were modified with polypyrrole-chitosan (PPy-CHI) conducting polymer composite. The polymer layer was deposited on the gold surface by electrodeposition. This optical sensor was used for monitoring toxic metal ions with and without sensitivity enhancement by chitosan in water samples. The higher amounts of resonance angle unit (ΔRU) were obtained for PPy-CHI film due to a specific binding of chitosan with Pb^2+^ and Hg^2+^ ions. The Pb^2+^ ion bind to the polymer films most strongly, and the sensor was more sensitive to Pb^2+^ compared to Hg^2+^. The concentrations of ions in the parts per million range produced the changes in the SPR angle minimum in the region of 0.03 to 0.07. Data analysis was done by Matlab software using Fresnel formula for multilayer system.

## Introduction

In recent years, surface plasmon resonance (SPR) has been widely demonstrated to be an effective optical technique for in situ investigation of the optical and electrical properties of conducting polymers, such as polypyrrole films [1]. Surface plasmon resonance (SPR) is a quantum optical-electrical phenomenon arising from the interaction of light with a metal surface [2]. The charge density oscillations that exist at a metal-dielectric (or metal/vacuum) interface are called surface plasmons that propagate in a direction parallel to the metal-dielectric interface and are on the boundary of the metal and the external medium (air or water for example) [3]. So that, these oscillations are very sensitive to any change of this boundary, such as adsorption of molecules to the metal surface.

Heavy metals such as mercury and lead ions have long been recognized as a harmful environmental pollutant. Lower levels of lead can affect on the central nervous system, kidney, and blood cells [4] and in severe cases can cause convulsions, coma, and even death. Lead interferes with the development of the nervous system and is therefore particularly toxic to children and unborn babies, causing potentially permanent learning and behaviour disorders [5].

There are many techniques for analysis trace metal e.g., atomic absorption, atomic emission, and fluorescence spectrometry, inductively coupled plasma-mass spectrometry (ICP-MS), and electrochemical techniques (such as ion-selective potentiometry and anodic stripping voltammetry) [6]. In contrast to their attractive analytical “figures of merit”, each of these techniques suffers from some certain disadvantages. Applications of all of these methods require a knowledge of chemistry and instrumentation and need exactitude apparatuses [7].

A major disadvantage of ICP-MS is the high capital cost of the instrumentation. In addition, this instrument is bulky and not selective to different charge states of an element. The non linearity of the calibration curves is a disadvantage of the atomic absorption spectroscopy (AAS) technique (in the absorbance range higher than 0.5 to 1). Running and investment costs of inductively coupled plasma atomic emission spectroscopy (ICP-AES) are high. Another disadvantage of ICP-AES is spectral interferences (due to many emission lines). Voltammetric methods are simple, inexpensive, and portable but they suffer of interferences inherent in complex sample matrix. Anodic stripping voltammetry can only measure amalgam-forming metal species [8]. Thereby, the complementary methods have developed to overcome some of these shortcomings.

SPR is a method that provides an effective optical technique to detect monolayer thicknesses of the material on the conducting surface. This technique exhibits a good sensitivity, stability, and reproducibility; sometimes even changes in refractive index of approximately 10^−5^ can be detected by SPR technique [9]. Conducting sensors based on PPy film with different dopant were developed for detecting volatile aromatic hydrocarbons [10] and volatile organic solvent sensors have also been fabricated using PPy films on conducting glass substrates [11]. Selective SPR sensors based on PPy have been reported for the detection of Cu^2+^ [12], and also for biosensor such as pseudomonas aeruginosa cells [13].

In previous work [14], we obtained the refractive indexes of PPy and PPy-CHI thin films (in contact with air) by SPR technique. Since they produced a sharp peak of resonance angle, it was shown these polymers are capable for using in sensitive optical sensors.The aim of the current study is to prepare optical sensors based on PPy and PPy-CHI composite films to detect trace amount of Hg^2+^ and Pb^2+^. The optical sensors were used for monitoring of toxic metal ions with and without sensitivity enhancement by chitosan. To our knowledge, this is the first report of a SPR sensor that uses PPy-CHI biocomposite for heavy metal detection.

## Materials and Methods

Pyrrol monomer (Acros Organic) was distilled and stored below 4°C. All the other reagents including chitosan (a local company with 88% degree of deacetylation) and *p*-toluene sulfonate (Fluka) were of analytical grade,and used without further purification. All of the electropolymerization of pyrrole was done via a typical three-electrode electrochemical cell arrangement. A saturated calomel electrode (SCE) and a carbon rod were used as reference and counter electrode, respectively. The thin layer of gold covered onto the glass slide was used as working electrode.

The glass microscope slides (ZF52, n = 1.8395) were cleaned using HCl/H_2_O at 60°C for 15 min following by rinsing the surface with deionized water and drying at 50°C. Each glass slide was coated with a thin layer of gold, using a sputtering coating (Model SC7940, Polaron). The thin layer of gold coated onto glass slide was used as working electrode in electrochemical deposition of polymer.

The sample of PPy-CHI was prepared in a solution containing 0.015 M Pyrrole, 0.005 M *P*-TS and 0.07% (w/v) chitosan in a 1% acetic acid solution. The PPy film was prepared, using the same composition of pyrrole and *P*-TS without chitosan. Electrochemical deposition of PPy-CHI and PPy films on the gold substrate was performed using a potentiostat (Model: PS 605, USA), imposing a constant potential of 0.85 V (vs SCE) for 150 sec. The results from FT-IR, electrical conductivity, and XRD study confirmed the incorporation of chitosan in PPy structure [15]. The electrochemical technique which can be used to electropolymerization can be chosen from all known techniques such as; potentiostatic, galvanostatic and potential/ current sweep, etc.

**Figure 1:**
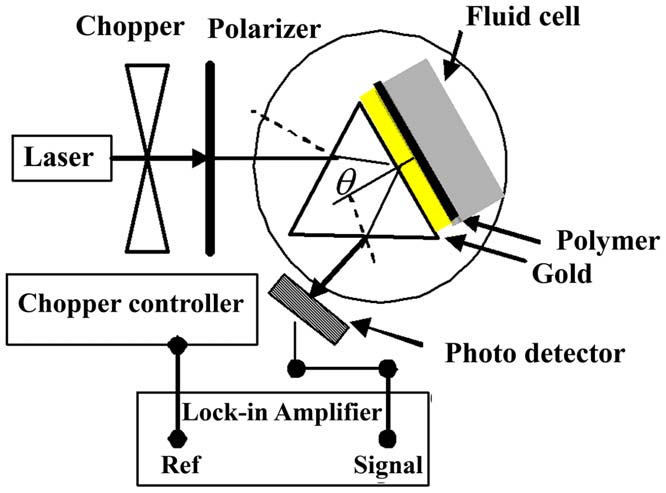
Set up configuration for SPR measurements.

The surface of the polymer films was washed with distilled deionized water (DDW). The thin slides coated with polymer were attached to the prism with high index glass (ZF52, n = 1.8395) by using index-matching fluid (n = 1.52 new port company). SPR measurements were conducted with a set up assembled according to the Kretschmann's prism configuration (Figure 1). The precision of rotator stage was 0.003. Data analysis was done by Matlab software using Fresnel formula for multilayer system [16]. In the Fresnel equation for *p*-polarized light the reflectivity of the light (*R*(*θ*)) is a function of angle and wavelength where *θ* is the angle of incidence light at the interface between the prism and surface [17]. DDW was then run on the surface of the films to establish a baseline. When a stable baseline of the SPR angle was obtained, the PPy and PPy-CHI film exhibited a SPR angular profile deep enough for precise definition of the resonance angle. The refractive indexes of the polymer films were successfully measured by SPR technique.

## Results and Discussion

**Figure 2:**
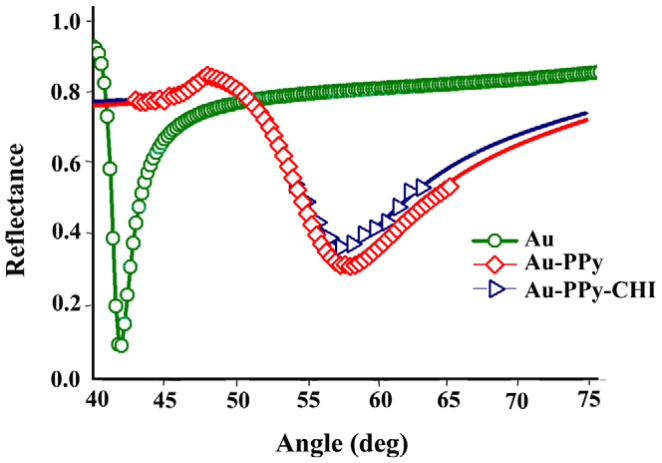
SPR angular profiles for DDW on the Au, Au-PPy and Au- PPy-CHI surface.

Figure 2 shows the surface plasmon resonance (SPR) reflectivity curves for the glass/Au/DDW, (Au), and glass/Au/PPy/DDW, (Au-PPy), and glass/Au/PPy-CHI/DDW, (Au-PPy-CHI), systems. The values of the real and imaginary parts of the refractive indexes of films were successfully obtained by nonlinear least square fitting using Fresnel equations for multilayer system. The resonance angle (*θ*spr) and the optical constant for the PPy film were 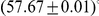 and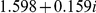, respectively. The formation of a thin film of polypyrrole with chitosan on the gold surface was monitored as a decreasing SPR angle for the PPy-CHI film.

The resonance angle (*θ*spr) and the optical constant of the PPy-CHI film were 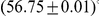 and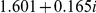, respectively. The dip widths of the curves for the PPy-CHI and PPy films were larger than Au/DDW, which was proportional to the attenuation of the surface plasmon. The thickness of the thin films was calculated using the Fresnel equation and the values of 26 nm and 28 nm were obtained for the PPy-CHI and PPy thin films, respectively. When a stable baseline of SPR angle was obtained for PPy and PPy-CHI films, the surface of the films were ready for binding tests for Hg^2+^ and Pb^2+^ samples.

### Binding of Hg^2+^ and Pb^2+^ on to PPy and PPy-CHI films

Different concentrations of Hg^2+^ and Pb^2+^ in aqueous solution were injected in to the fluid cell attached to the films, and the SPR angle (resonance units = RU) was monitored to detect any binding interactions. The increase in the SPR angle (ΔRU) for each Hg^2+^ and Pb^2+^ concentration was determined based on the resonance angle of the ions (after the ion binding event) and the DDW baselines before running ions samples over a time duration of Δ*t*. These ΔRU results indicated that Hg^2+^ and Pb^2+^ were binding successfully with the PPy and PPy-CHI. Each sample was run for 20 min. The concentrations of ions in the parts per million range produced the changes in the SPR angle minimum in the region of 0.03 to 0.07. Due to the high sensitivity of SPR, it also is possible to detect concentrations in the parts per billion ranges. A control experiment for the Hg^2+^ and Pb^2+^ ions with bare Au surface was done for 30 min, which produced no changes in the resonance angle indicating that the polymer was necessary to react with the ions. After exposure to each sample concentration, all of the PPy and PPy-CHI films were washed with DDW to establish a baseline, as the history of the films was irrelevant.

### Sensitive optical sensor for Hg^2+^ and Pb^2+^detection

**Figure 3:**
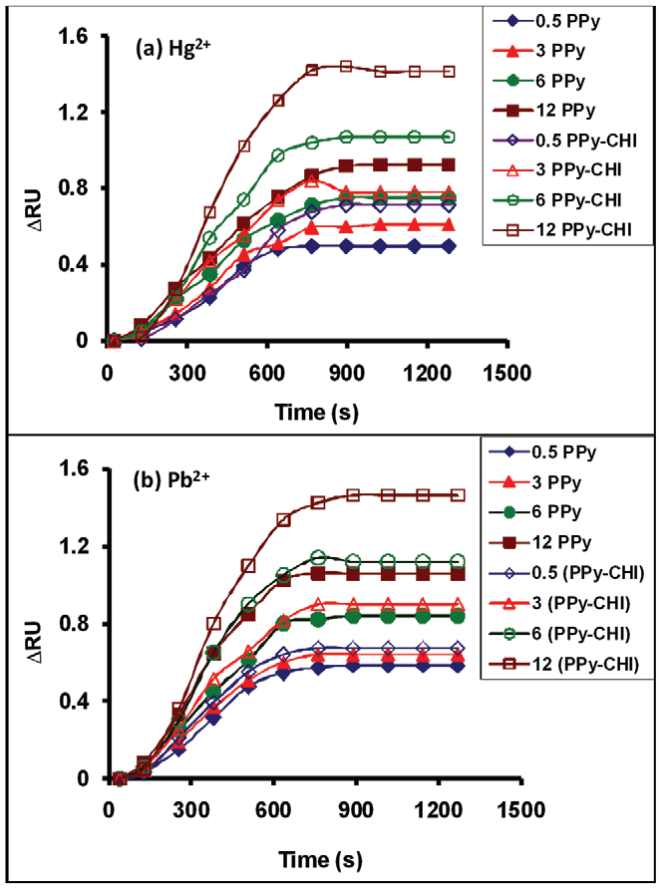
The sensograms for 0.5–12 ppm ion binding on PPy and PPy-CHI surface. Filled symbols (PPy surface), Open symbols (PPy-CHI surface). SPR sensogram for (a) Hg^2+^ and (b) Pb^2+^.

The increase in the SPR angle for each Hg^2+^ and Pb^2+^ concentration indicated that these ions were binding successfully with PPy. The typical sensograms for 0.5–12 ppm Hg^2+^ and Pb^2+^ binding on PPy are presented in Figure 3. It can be observed that, first, ΔRU increased over the time for each sample, but after almost 700 sec the graphs showed a constant value. This result can be explained by saturation of the binding sites available on the PPy surface.

It seems that there is a chemical binding of ions in water with the PPy thin film that was immobilized on the gold surface. The interaction between Hg^2+^ and PPy was also reported by Yu *et al*. [9], as the natural preference for a combination of soft acid (Hg^2+^) and soft base (PPy).

On the other hand, there are some studies on the treatment of solutions of metal ions with redox polymers, such as polypyrroles, polyanilines and polythiophenes, to reduce the ions to a lower valence [18]. These literatures show that PPy can interact with certain heavy metals through an acid and base interaction or an oxidation reaction.

### Sensitivity Enhancement Using CHI

Chitosan was used to prepare the PPy-CHI composite film to function as a sensitivity enhancement agent for detecting for heavy metals ions. DDW was run to establish a baseline. The decreasing SPR angle for the PPy-CHI film when compared to the PPy film indicated that chitosan incorporated inside the PPy structure. The sensograms for 0.5–12 ppm Hg^2+^ and Pb^2+^ binding on the PPy-CHI are also presented in Figure 3. The composite films of PPy-CHI showed the higher ΔRU compared to the PPy film for each time and concentration, indicating a higher sensitivity of the PPy in the presence of chitosan. In fact, the natural biopolymers have adsorption properties toward certain heavy metals. Of the many absorbents identified, chitosan has the highest sorption capacity for several metal ions [19]. Following this interaction of CHI and metal ions to enhance the original ΔRU, lower concentrations of ions could be readily detected with less interference.

**Figure 4:**
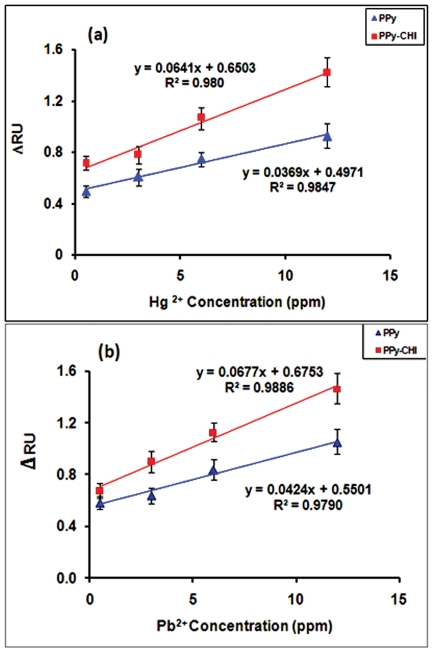
Standard calibration curves of ΔRU versus ion concentration for PPy and PPy-CHI films. (a) Hg^2+^ and (b) Pb^2+^.

In order to show the sensitivity of the PPy and PPy-CHI films for metal ions, standard calibration curves were plotted for sensors and are shown in Figure 4. Different concentrations of ions were run for 20 min and all ΔRU were taken from the first 13 min of each binding curves. Each concentration was repeated 3 times and the standard deviation data were in the region of 0.06 to 0.18 and it is shown as error bar on the diagram of sensitivity.

These figures clearly show the enhancement of sensitivity for sample determination in the presence of chitosan. In the both sensograms the SPR dip shift is directly correlated with the ions concentration ([Hg^2+^] or [Pb^2+^]). Linear regression analysis of the sensors yielded the following equations with all correlation coefficients *R* ^2^ greater than 0.97, revealing good linearity for the relationship between the SPR dip shift and ions concentration:
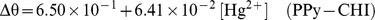


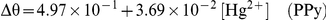


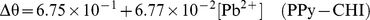


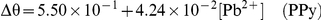



The slop of the standard calibration curve in the PPy-CHI sensor was almost 2 times higher than that for the PPy sensor without chitosan. It can be seen that all the standard calibration curves showed a significant deviation from 0. This deviation was due to saturatation of the binding sites available on the polymer surface, a fundamental limitation of maximum uptake capacity [20].

### Conclusion

The binding interactions of heavy metals with PPy and CHI on the gold surface were monitoring using surface plasmon resonance technique. This technique was used to fabricate the PPy and PPy-CHI optical sensors for monitoring trace amount of Hg^2+^ and Pb^2+^. The higher **Δ**RU results are due to specific binding of chitosan with Hg^2+^ and Pb^2+^ ions. Pb^2+^ ion binds to the polymer films most strongly and the sensors (based on PPy and PPy-CHI thin films) are more sensitive to Pb^2+^ compared to the Hg^2+^. The refractive indexes of the polymer films were successfully measured by SPR technique.
